# In Vitro α-Glucosidase and Dipeptidyl Peptidase-IV Inhibitory Activities of Ethanol Extract and Its Fractions from the Leaves and Stems of *Glehnia littoralis*

**DOI:** 10.3390/foods15213980

**Published:** 2026-11-06

**Authors:** Jaemin Choi, Joong-Hyuck Auh

**Affiliations:** Department of Food Science and Biotechnology, Chung-Ang University, Anseong 17546, Republic of Korea; cjm9657@naver.com

**Keywords:** *Glehnia littoralis*, leaves and stems, α-glucosidase, dipeptidyl peptidase-IV (DPP-IV), metabolomics, rutin, quercetin 3-β-D-glucoside (Q3G), parallel reaction monitoring (PRM)

## Abstract

This study evaluated the in vitro α-glucosidase and dipeptidyl peptidase-IV (DPP-IV) inhibitory and antioxidant activities of *Glehnia littoralis* leaves and stems, which are traditionally consumed but less studied than the roots. The 70% ethanol extract was partitioned into three fractions (F1, F2, F3). Fraction 2 (F2) concentrated total phenolics (234.71 mg GAE/g) and flavonoids (155.61 mg QE/g) most effectively, showing strong DPPH radical scavenging activity (IC_50_ = 32.09 µg/mL). F2 also demonstrated dual-targeting inhibitory efficacy against α-glucosidase (IC_50_ = 314.41 µg/mL) and DPP-IV (IC_50_ = 209.67 µg/mL), comparable to acarbose in α-glucosidase inhibition (yeast enzyme assay), though much less potent than sitagliptin against DPP-IV. Exploratory non-targeted metabolomics highlighted rutin and quercetin 3-β-D-glucoside (Q3G) as discriminating markers (VIP ≥ 2.0); their contents in F2, estimated by Parallel Reaction Monitoring, were 188.97 mg/g and 43.44 mg/g, respectively. In a concentration-addition analysis, the summed dose fractions of rutin, Q3G, and chlorogenic acid at the IC_50_ of F2 were approximately 0.32 and 0.62 for α-glucosidase and DPP-IV, respectively, suggesting contributions from additional, untested constituents such as kaempferol-3-*O*-rutinoside. These findings indicate that *Glehnia littoralis* leaves and stems merit further investigation as potential functional food resources for blood glucose regulation, pending cell-based and in vivo validation.

## 1. Introduction

Diabetes mellitus is a critical global health challenge characterized by chronic metabolic disruption and persistent hyperglycemia [1]. The International Diabetes Federation estimates that the global diabetic population will reach 853 million by 2050, with Type 2 diabetes mellitus (T2DM) accounting for over 90% of cases [2]. A key therapeutic target in managing T2DM is the control of postprandial (after-meal) blood glucose spikes, which are directly linked to oxidative stress and severe cardiovascular complications [3]. Inhibiting α-glucosidase—the primary intestinal enzyme responsible for carbohydrate digestion—and suppressing dipeptidyl peptidase-IV (DPP-IV) to prolong the activity of blood-sugar-regulating incretin hormones represent two of the most effective strategies to stabilize postprandial glucose levels [4,5].

A wide spectrum of synthetic oral antidiabetic drugs, such as metformin, sulfonylureas, and commercial DPP-IV inhibitors, are clinically established; however, their long-term administration is frequently constrained by adverse effects ranging from gastrointestinal distress to severe systemic risks like lactic acidosis [6,7]. Consequently, there is a strong shift in both pharmaceutical and nutritional sciences toward identifying safer, multi-target bioactive compounds from natural sources [8]. Flavonoids in particular have been extensively documented as dual-target inhibitors capable of simultaneously suppressing carbohydrate-hydrolyzing and incretin-degrading enzymes, positioning them as attractive candidates for multi-target dietary intervention in T2DM management [9,10]. Halophytes—plants naturally adapted to high-saline environments—have emerged as promising candidates due to their evolutionary adaptation to accumulating dense concentrations of protective secondary metabolites, particularly antioxidant phenolic compounds [11].

*Glehnia littoralis* is a representative halophyte distributed across East Asian and North American coastlines, traditionally utilized as both a medicinal herb and a dietary vegetable [12]. The sprouting leaves of this species have also been consumed as vegetables in Korea and Japan, and recent studies have examined the chemical composition and physiological properties of its aerial tissues, including furanocoumarin and polyacetylene profiling [13], antibacterial and antioxidant enhancement via lactic acid bacteria fermentation [14], skin-whitening and anti-wrinkle effects of leaf extracts [15], and antioxidant and anti-inflammatory activity of leaf-derived phenolics in macrophage models [16]. A comparative regional analysis further demonstrated compositional variation in phenolic content across different plant parts and geographic origins of *Glehnia littoralis* [17], underscoring the need for a more systematic, tissue-specific investigation of its bioactive potential. To date, mainstream research has heavily prioritized its underground roots, leaving a distinct gap in our understanding of its aerial parts (leaves and stems). While a few metabolic profiling studies have identified general terpenoids and coumarins in these aerial structures [18,19], their α-glucosidase and DPP-IV inhibitory activities have not yet been systematically evaluated. This creates a significant bottleneck for research, as the leaves and stems are increasingly cultivated as food crops.

To address this gap, this study comprehensively investigated the α-glucosidase and DPP-IV inhibitory activities of *Glehnia littoralis* leaf and stem ethanol extracts. Active metabolites were tracked through preparative high-performance liquid chromatography (prep-HPLC) fractionation and systematically identified using ultra-high-performance liquid chromatography–Orbitrap tandem mass spectrometry (UHPLC-Orbitrap MS/MS) combined with multivariate statistical analysis. Furthermore, target active markers were quantified using the parallel reaction monitoring (PRM) mode. Crucially, our findings demonstrate that specific fractions of *Glehnia littoralis* aerial parts possess potent, dual-targeting enzyme inhibitory efficacy, positioning these underutilized agricultural byproducts as potential materials for functional foods.

## 2. Materials and Methods

### 2.1. General Materials

Folin & Ciocalteu’s phenol reagent (2 M), gallic acid, aluminum chloride (AlCl_3_), sodium nitrite (NaNO_2_), quercetin hydrate, quercetin 3-β-D-glucoside, rutin hydrate, chlorogenic acid, 4-nitrophenyl α-D-glucopyranoside (pNPG), and a dipeptidyl peptidase-IV (DPP-IV) inhibitor screening kit were purchased from Sigma-Aldrich (St. Louis, MO, USA). 2,2-Diphenyl-1-picrylhydrazyl (DPPH) was obtained from Alfa Aesar (Haverhill, MA, USA). α-Glucosidase from *Saccharomyces cerevisiae* was purchased from Megazyme (Wicklow, Ireland). Yeast-derived α-glucosidase was employed, consistent with its widespread use in the screening of plant-derived antidiabetic compounds [20]. Whatman No. 1 qualitative filter paper and 0.2 µm polyvinylidene fluoride (PVDF) syringe filters were obtained from GE Healthcare Life Sciences (Little Chalfont, UK). Liquid chromatography (LC) grade acetonitrile was acquired from Thomas Scientific (Swedesboro, NJ, USA). Sodium hydroxide (NaOH) was purchased from Samchun Chemical (Pyeongtaek, Republic of Korea). Extra pure (EP) grade ethanol, anhydrous sodium carbonate (Na_2_CO_3_), and potassium phosphate monobasic were obtained from Duksan (Ansan, Republic of Korea). Ascorbic acid and anhydrous potassium phosphate dibasic were purchased from Daejung (Siheung, Republic of Korea). Mass spectrometry (MS) grade water, formic acid, and acetonitrile for ultra-high-performance liquid chromatography–Orbitrap tandem mass spectrometry (UHPLC-Orbitrap MS/MS) analysis were purchased from Thermo Fisher Scientific (Waltham, MA, USA).

### 2.2. Preparation of Glehnia littoralis Extract

*Glehnia littoralis* cultivated in Pohang-si, Gyeongsangbuk-do, South Korea, was used in this study. Fresh stems and leaves of *Glehnia littoralis* were freeze-dried at −50 °C for 72 h and subsequently ground using a commercial blender (UNB-A9001, Unix, Ningbo, China). A 3 g aliquot of the dried sample was mixed with 150 mL of 70% ethanol (1:50, *w*/*v*) and subjected to ultrasonic-assisted extraction on ice for 3 cycles using a Sonics^®^ Vibra-Cell™ VCX 750 ultrasonic unit (Sonics & Materials Inc., Newtown, CT, USA). Each extraction cycle consisted of 5 min of sonication at 60% amplitude, with pulsing every 3 s at 10 s intervals. Following sonication, the mixture was centrifuged at 15,000× *g* for 30 min, and the supernatant was filtered through Whatman No. 1 qualitative filter paper. The filtrate was concentrated using a rotary vacuum evaporator (Eyela, Tokyo, Japan) at 36 °C and lyophilized to yield a dried crude extract powder. The obtained powder was stored in a freezer at −50 °C until further experimentation.

### 2.3. Fractionation of Glehnia littoralis Extract

The *Glehnia littoralis* ethanolic extract was fractionated using a preparative high-performance liquid chromatography (prep-HPLC) apparatus (LC-Forte/R, YMC, Kyoto, Japan). Chromatographic separation was carried out on a YMC Triart C18-S column (250 × 15 mm, 20 µm particle size) with a mobile phase consisting of water (A) and acetonitrile (B) at a flow rate of 10 mL/min. The injection volume was 5 mL at a sample concentration of 15 mg/mL. Elution was monitored using a diode array detector (DAD) at wavelengths of 210, 254, and 280 nm. The gradient elution program was optimized as follows: 0–7 min, 0% B; 7–8 min, linear transition from 0% to 40% B; 8–16 min, 40% B; 16–30 min, linear gradient from 40% to 100% B; and 30–42 min, 100% B. Fractions were collected periodically based on the target peaks and dried for bioactivity evaluation.

### 2.4. Measurement of Total Phenolic and Flavonoid Contents

The total phenolic content (TPC) of the *Glehnia littoralis* extract and its fractions was determined using Folin & Ciocalteu’s phenol reagent according to a modified colorimetric method [21]. Briefly, a 100 µL aliquot of the sample (1 mg/mL) was mixed with 500 µL of 0.2 M Folin & Ciocalteu’s reagent and 400 µL of 10% (*w*/*v*) Na_2_CO_3_. The mixture was incubated in a water bath at 37 °C for 1 h, and the absorbance was measured at 750 nm using a nanophotometer (IMPLEN, München, Germany). TPC was expressed as milligrams of gallic acid equivalents per gram of dry weight (mg GAE/g dry weight of sample).

The total flavonoid content (TFC) was evaluated using an aluminum chloride colorimetric method [22]. A 100 µL portion of the sample (1 mg/mL) was blended with 400 µL of distilled water, followed by the addition of 30 µL of 5% (*w*/*v*) NaNO_2_. After a 5 min incubation at room temperature, 30 µL of 10% (*w*/*v*) AlCl_3_ was added and allowed to react for 6 min. Finally, 200 µL of 1 M NaOH and 240 µL of distilled water were added to the mixture. The absorbance was immediately recorded at 415 nm using the nanophotometer. TFC was calculated and expressed as milligrams of quercetin equivalents per gram of dry weight (mg QE/g dry weight of sample).

### 2.5. Evaluation of Antioxidant Activity

The DPPH free radical scavenging activity was determined using a modified colorimetric protocol [23]. The extract and fractions were dissolved in DMSO or distilled water at 100 mg/mL and sequentially diluted to various concentrations with methanol. Ascorbic acid was utilized as the reference standard. A 200 µL aliquot of the sample or standard solution was mixed with 100 µL of 0.2 mM DPPH solution in a 96-well plate and incubated in the dark at 37 °C for 30 min. The absorbance was recorded at 517 nm using a microplate reader (Molecular Devices, San Jose, CA, USA). The DPPH radical scavenging activity was calculated as a percentage relative to the control blank.

(1) DPPH radical scavenging activity (%) = 100 − (A_sample_/A_control_ × 100)

where A_control_ is the absorbance of the DPPH solution without sample, and A_sample_ is the absorbance of the reaction mixture containing the sample or standard. To compare the antioxidant capacities, the results were expressed as the half-maximal inhibitory concentration (IC_50_ value, µg/mL), representing the concentration required to scavenge 50% of the DPPH free radicals.

### 2.6. α-Glucosidase and DPP-IV Inhibitory Activities

#### 2.6.1. α-Glucosidase Inhibitory Activity

The α-glucosidase inhibitory activity was measured via a modified colorimetric method [20]. In a 96-well plate, 32 µL of the sample extract or fraction at various concentrations was pre-incubated with 40 µL of α-glucosidase enzyme solution (0.8 U/mL) and 208 µL of 50 mM potassium phosphate buffer (pH 6.8) at 37 °C for 10 min. The enzymatic reaction was initiated by adding 40 µL of 2.5 mM pNPG substrate. The absorbance was continuously monitored at 405 nm at 1 min intervals for a total of 30 min at 37 °C. The final volume was 320 µL, and sample concentrations are expressed as final concentrations in the reaction mixture. The enzyme inhibition percentage was calculated using the following equation:

(2) Inhibition (%) = [1 − (A_sample_ − A_sample_blank_)/(A_control_ − A_control_blank_)] × 100

where *A*_control_ is the absorbance of the 100% enzyme reaction without inhibitor, *A*_control_blank_ is the absorbance of the buffer and substrate blank, *A*_sample_ is the absorbance of the complete sample reaction, and *A*_sample_blank_ is the absorbance of the sample mixture without the enzyme. To compare inhibitory potency, the samples were evaluated across multiple concentrations to determine their IC_50_ values (µg/mL).

#### 2.6.2. DPP-IV Inhibitory Activity

The DPP-IV inhibitory activity was determined using a commercial DPP-IV inhibitor screening kit (MAK203, Sigma-Aldrich) according to the manufacturer’s instructions. A 25 µL aliquot of the sample solution prepared at 4-fold the testing concentration was mixed with 50 µL of the inhibition reaction mix (comprising 49 µL of assay buffer and 1 µL of DPP-IV enzyme) in a 96-well black plate. The mixture was pre-incubated at 37 °C for 10 min. Subsequently, the enzymatic reaction was initiated by adding 25 µL of the substrate solution (23 µL of assay buffer mixed with 2 µL of DPP-IV substrate). The fluorescence intensity (Ex/Em = 360/460 nm) was monitored kinetically every minute for 30 min at 37 °C using a microplate reader.

The relative DPP-IV inhibition percentage was calculated based on the slopes of the linear reaction range between 15 min (*T*_1_) and 30 min (*T*_2_):

(3) Slope = (Fluorescence at *T*_2_ − Fluorescence at *T*_1_)/(*T*_2_ − *T*_1_)

(4) Inhibition (%) = [1 − (Slope_sample_/Slope_control_)] × 100

Similar to the other bioassays, the sample concentrations were varied to calculate and compare the IC_50_ values (µg/mL) for evaluating relative potency.

### 2.7. UHPLC-Orbitrap MS/MS Profile Analysis

Qualitative profiling and quantitative analysis of the *Glehnia littoralis* extract and fractions were performed using a Vanquish UHPLC system equipped with a DAD and coupled with a Q Exactive™ Orbitrap mass spectrometer (Thermo Fisher Scientific, Waltham, MA, USA). Chromatographic separation was achieved at 30 °C on a Thermo Hypersil Gold aQ C18 column (100 × 2.1 mm, 1.9 µm particle size). The mobile phase consisted of 0.1% formic acid in water (A) and 0.1% formic acid in acetonitrile (B). The optimized gradient program was operated as follows: 0–5 min, 5% B; 5–16 min, linear gradient to 100% B; 16–20 min, 100% B; 20–21 min, linear transition to 5% B; and 21–24 min, 5% B. The flow rate was maintained at 0.3 mL/min, and the injection volume was 5 µL. The UV-Vis spectrum was monitored from 190 to 800 nm.

Mass spectrometry was conducted using a heated electrospray ionization (HESI) source operated in the positive (POS) and negative (NEG) ionization modes in separate runs. The capillary temperature was kept at 320 °C, and the spray voltage was 3.80 kV (POS) and 2.50 kV (NEG). The sheath gas, auxiliary gas, and sweep gas flow rates were set at 40 mL/min, 10 mL/min, and 1 L/min, respectively, with an S-lens RF level of 50.0%. Full MS scans combined with parallel reaction monitoring (PRM) scans were acquired over a mass-to-charge ratio scan range of *m*/*z* 100 to 1500. Total ion chromatograms (TIC) were visualized and evaluated using Xcalibur 4.1 software (Thermo Fisher Scientific). Prior to analysis, all samples were filtered through a 0.2 µm PVDF syringe filter.

### 2.8. Data Processing, Metabolite Identification, and Statistical Analysis

The raw UHPLC-Orbitrap MS/MS datasets acquired in negative ionization mode were processed using Compound Discoverer 3.1 software (Thermo Fisher Scientific). The data processing criteria were established with a precursor mass range of 100–1500 Da, an alignment mass tolerance of 5 ppm, a retention time (RT) range of 0–20 min, a peak merging RT tolerance of 0.2 min, a signal-to-noise ratio threshold (S/N) greater than 3, and a minimum peak intensity of 10^5^. Metabolites were tentatively identified using their accurate precursor mass (MS^1^) and characteristic fragment ions (MS^2^) by cross-referencing with online databases, including mzCloud, PubChem, MassBank of North America (MoNA), and FooDB. Annotation confidence was assigned according to the Metabolomics Standards Initiative (MSI) [24]: Level 1, matched with an authentic standard by retention time and MS/MS spectrum; Level 2, putative annotation by accurate mass and MS/MS library matching; Level 4, unknown. Each sample was divided into three aliquots, each of which was injected once (*n* = 3 per group), consecutively by group within approximately 5 h. Solvent blanks (50% acetonitrile) were analyzed under the same conditions, and background features were removed using the Mark Background Compounds node. Peak areas were exported without further normalization, Pareto-scaled, and analyzed in SIMCA using the autofit function, in which the number of components is determined by cross-validation. Neither internal standards nor pooled QC samples were used. For multivariate statistical evaluation, the processed metabolomic data were imported into SIMCA 17.0 software (Umetrics, Umeå, Sweden). Principal component analysis (PCA) and orthogonal partial least squares-discriminant analysis (OPLS-DA) were performed following Pareto scaling to characterize and visualize chemical variances across the extracts and fractions. S-plots derived from OPLS-DA were utilized to identify distinctive potential biomarkers. The VIP score, which quantifies each variable’s contribution to the OPLS-DA model based on both loading weight and explained variance, was calculated according to the algorithm described by Trygg and Wold [25] and further refined for OPLS models by Galindo-Prieto et al. [26]; a VIP ≥ 1 is conventionally regarded as an initial screening threshold, and the more stringent VIP ≥ 2.0 applied here was adopted to prioritize the most robust discriminating variables. All bioassay results were derived from three independent replicates (n = 3) and expressed as mean ± standard deviation (SD). Statistical significance was assessed by one-way analysis of variance (ANOVA) followed by Duncan’s multiple range test at *p* < 0.05 using SPSS 12.0 software (IBM SPSS Statistics, Chicago, IL, USA). To evaluate the relative potency of the samples, IC_50_ values for α-glucosidase and DPP-IV inhibition of all samples, including the crude extract, fractions, pure compounds, and positive controls, were determined by the same procedure, log-probit analysis using SPSS 12.0, for each independent replicate. When 50% inhibition was not reached at the highest tested concentration, the IC_50_ was reported as greater than that concentration. IC_50_ values of pure compounds and positive controls were additionally expressed in µM using their molecular weights (rutin, 610.52; Q3G, 464.38; chlorogenic acid, 354.31; acarbose, 645.60; sitagliptin, 407.31 g/mol). IC_50_ values for DPPH radical scavenging were calculated from the concentration-response data of each replicate. Experimental graphs were plotted using GraphPad Prism 9.4 software (GraphPad Software Inc., San Diego, CA, USA).

### 2.9. Metabolite Quantification via PRM

Target active metabolites in the crude extract and the highest performing bioactive fraction were targeted for quantification using the PRM acquisition mode in negative ionization mode. Quantitative verification was executed based on precise precursor molecular weights (*m*/*z*) paired with their specific diagnostic MS^2^ product fragment intensities [27]. The target parameters utilized for quantification were as follows: rutin (*m*/*z* 609.1463, fragment *m*/*z* 300.0275); chlorogenic acid (*m*/*z* 353.0878, fragment *m*/*z* 191.0554); and quercetin 3-β-D-glucoside (*m*/*z* 463.0881, fragment *m*/*z* 300.0274). Blank-subtracted peak areas were quantified against external calibration curves of authentic standards. Because isotope-labeled internal standards were not available and recovery experiments were not performed, the reported contents should be regarded as estimates that may be affected by matrix effects. Among the biomarker candidates satisfying the VIP ≥ 2.0 threshold, rutin, chlorogenic acid, and Q3G were identified at MSI Level 1 by matching their retention times (ΔRT ≤ 0.03 min) and MS/MS fragmentation patterns with those of authentic standards and subsequently included in PRM-based quantification and pure-standard enzyme inhibition assays. Kaempferol-3-*O*-rutinoside, in contrast, was identified based on accurate mass and MS/MS fragmentation matching alone, without confirmation against an authentic reference standard, and was therefore treated as a putatively identified biomarker (MSI Level 2) that was not carried forward into quantitative and enzyme-inhibition validation in the present study. The contribution of the quantified compounds to the activity of F2 was estimated by a concentration-addition approach. The concentration of each compound *i* at the IC_50_ of F2 was estimated as C_i_ = w_i_ × IC_50_, F2/1000 (w*i*, PRM content in mg/g), and the summed dose fraction Σ(C_i_/IC_50,i_) was calculated using the IC_50_ of each pure compound. A value of 1 indicates that the compounds can fully account for the 50% inhibition by F2 under concentration addition, whereas a value below 1 indicates that they cannot; this index does not quantify the percentage contribution of each compound.

## 3. Results

### 3.1. Extraction and Fractionation Yields of Glehnia littoralis

The extraction of *Glehnia littoralis* leaves and stems using 70% ethanol yielded 36.60 ± 1.26% of the initial freeze-dried weight. To separate different chemical components, the crude extract was subjected to preparative high-performance liquid chromatography (prep-HPLC). Based on the UV chromatograms and visual color observations, the extract was systematically separated into three distinct fractions (F1, F2, and F3) corresponding to specific mobile phase gradient steps. F1 (Retention Time [RT] 0.5–13.0 min) was colorless and primarily eluted during the initial highly hydrophilic stage, which spanned the 0% B isocratic phase (0–7 min) and the beginning of the 40% B plateau (8–16 min). F2 (RT 13.0–27.2 min) exhibited a slight yellow color, eluting as the mobile phase transitioned through the remaining 40% B phase and advanced into the linear gradient from 40% to 100% B (16–30 min). Lastly, F3 (RT 27.2–42.0 min) displayed a distinct green coloration, elution during the final highly hydrophobic phase consisting of the end of the 40–100% B gradient and the 100% B washing step (30–42 min). The yields for F1, F2, and F3 were determined to be 84.32 ± 1.13%, 4.68 ± 0.39%, and 1.36 ± 0.01% of the total crude extract weight, respectively. The remaining fraction (~9.6% of the crude extract weight) was not recovered as a defined peak and likely reflects minor losses during collection and drying.

### 3.2. Total Phenolic and Total Flavonoid Content

The total phenolic content (TPC) and total flavonoid content (TFC) of the 70% ethanol extract and its three prep-HPLC fractions were evaluated (Table 1). Among all samples, F2 exhibited the highest TPC (234.71 ± 0.31 mg GAE/g) and TFC (155.61 ± 6.99 mg QE/g) values, significantly higher than those of the crude extract and the other fractions (*p* < 0.05). F3 showed an intermediate TPC (48.56 ± 1.04 mg GAE/g), whereas its TFC (20.44 ± 1.11 mg QE/g) was statistically comparable to that of the crude extract, while F1 exhibited the lowest phenolic and flavonoid concentrations (22.58 ± 0.45 mg GAE/g and 6.67 ± 0.15 mg QE/g), even lower than the crude 70% ethanol extract itself (38.43 ± 0.66 mg GAE/g and 21.47 ± 1.09 mg QE/g).

### 3.3. Antioxidant Activity

The antioxidant capacity of *Glehnia littoralis* extract and its fractions was measured via the DPPH radical scavenging assay (Table 1). All tested samples exhibited a concentration-dependent increase in radical scavenging capacity, and IC_50_ values were calculated to compare their relative efficacy. Among the experimental groups, F2 showed the lowest IC_50_ value (32.09 ± 2.52 µg/mL), indicating the highest antioxidant activity among the fractions, followed by the crude 70% ethanol extract (203.99 ± 4.25 µg/mL). In contrast, F1 and F3 exhibited significantly weaker radical scavenging properties, with IC_50_ values of 425.24 ± 17.72 µg/mL and 463.91 ± 4.68 µg/mL, respectively. The positive control, ascorbic acid, showed the lowest IC_50_ value overall (4.01 ± 0.06 µg/mL), as expected for a pure reference antioxidant compound.

### 3.4. α-Glucosidase and DPP-IV Inhibitory Activities

**Figure 1:**
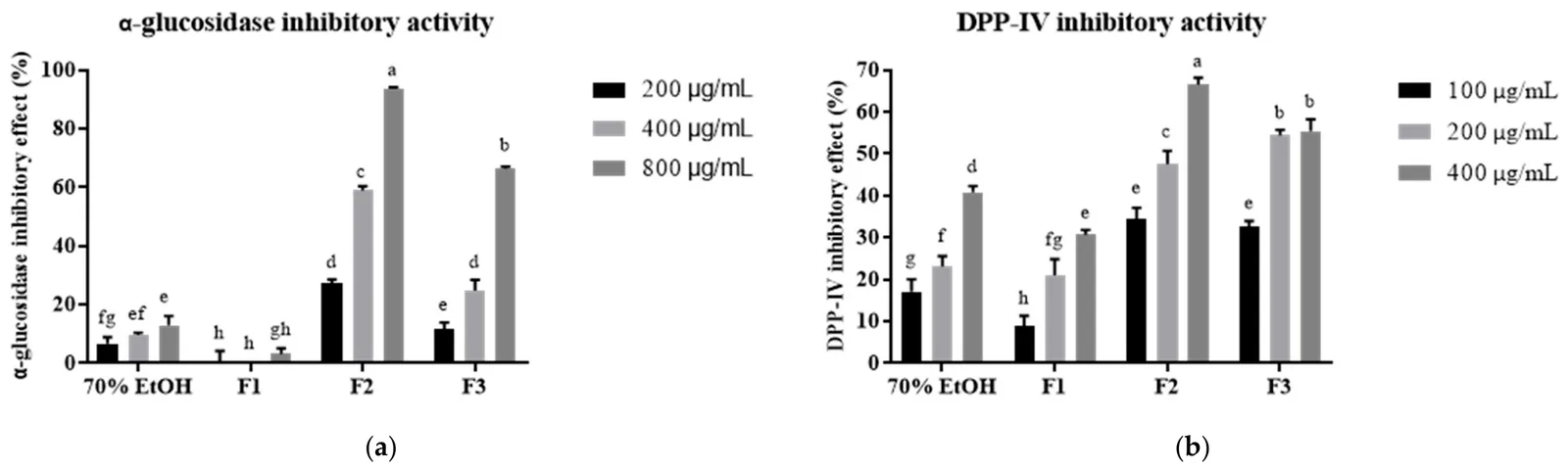
In vitro α-glucosidase and DPP-IV inhibitory activities of *Glehnia littoralis* 70% ethanol extract and its prep-HPLC fractions (F1, F2, and F3). (**a**) α-Glucosidase inhibitory activity at 200, 400, and 800 µg/mL; (**b**) DPP-IV inhibitory activity at 100, 200, and 400 µg/mL. Data are presented as mean ± SD (n = 3). Different letters (a–h) above the bars indicate statistically significant differences (*p* < 0.05) by Duncan’s multiple range test within each panel. Concentrations tested beyond the ranges shown were used for IC_50_ estimation but are not displayed: F1 at 1600 µg/mL in the α-glucosidase assay (12.42 ± 1.75%), and the crude extract at 800 µg/mL (59.87 ± 2.33%) and F1 at 800 and 1600 µg/mL (59.85 ± 2.40% and 74.92 ± 2.19%) in the DPP-IV assay.

To evaluate their enzyme inhibitory activities, the inhibitory effects against α-glucosidase and dipeptidyl peptidase-IV (DPP-IV) were measured across multiple concentrations for the crude 70% ethanol extract and its three prep-HPLC fractions (Figure 1a,b).

In the α-glucosidase inhibition assay (Figure 1a), all samples showed a concentration-dependent increase in inhibitory activity. At the highest tested concentration (800 µg/mL), F2 exhibited the greatest inhibitory effect (93.82%), followed by F3 (66.40%), while the crude extract and F1 showed markedly weaker activity (12.96% and 10.54%, respectively), with F2 significantly outperforming the other samples at every tested concentration (*p* < 0.05). IC_50_ values were calculated by probit analysis (Section 2.8). F2 showed the lowest IC_50_ (314.41 ± 6.78 µg/mL), followed by F3 (603.48 ± 25.80 µg/mL), and the IC_50_ of F2 was comparable to that of acarbose (422.16 ± 57.02 µg/mL) under the present assay conditions using yeast α-glucosidase. The crude extract and F1 did not reach 50% inhibition at the highest tested concentrations (IC_50_ > 800 and > 1600 µg/mL, respectively), consistent with their minimal inhibitory effects observed across all tested concentrations in Figure 1a.

For DPP-IV inhibition (Figure 1b), a similar concentration-dependent pattern was observed. F2 again emerged as the most active sample, showing the highest inhibition percentage (66.56%) at 400 µg/mL, compared with 55.36%, 40.78%, and 30.64% for F3, the crude extract, and F1, respectively, at the same concentration (*p* < 0.05). Based on dose–response curves fitted by log-probit regression, the IC_50_ value of F2 was calculated to be 209.67 ± 3.74 µg/mL. The remaining fractions and the crude extract exhibited higher IC_50_ values in the following order: F3 (239.66 ± 20.75 µg/mL), 70% EtOH crude extract (571.12 ± 41.62 µg/mL), and F1 (642.90 ± 49.38 µg/mL). The commercial DPP-IV inhibitor sitagliptin, used as a positive control, displayed an IC_50_ value of 0.0062 ± 0.0006 µg/mL (6.22 ng/mL).

### 3.5. Metabolomic Analysis by Multivariate Statistical Analysis

**Figure 2:**
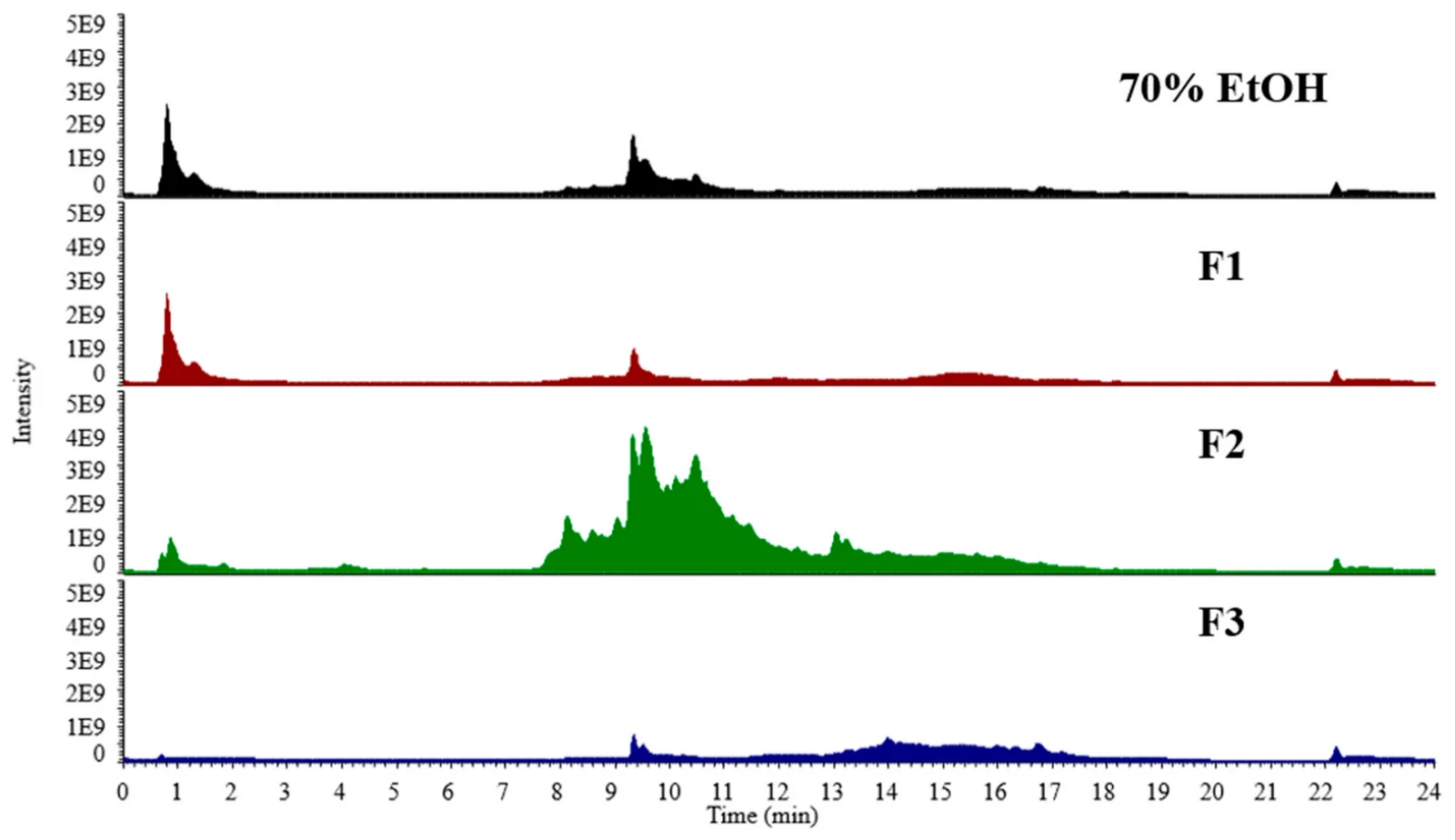
Total ion chromatograms (TIC) of *Glehnia littoralis* 70% ethanolic extract and its prep-HPLC fractions (F1, F2, and F3) obtained in negative-ion (NEG) mode.

Metabolomic analysis of *Glehnia littoralis* ethanolic extract and its fractions was conducted using UHPLC-Orbitrap MS/MS. Data were acquired in both POS and NEG modes. In POS mode, PCA explained 84.8% of the total variance, and F2 was clearly separated from the other samples (Figure S1); because rutin and Q3G showed higher VIP values in NEG mode (5.19 and 2.93) than in POS mode (1.93 and 1.69), subsequent analyses focused on NEG-mode data. Figure 2 illustrates the negative-mode total ion chromatogram (TIC) of the extract and its fractions. Based on the peak intensities and elution patterns obtained using the solvent gradient conditions, it was anticipated that F1 would contain relatively more hydrophilic compounds eluted in the early phase, while F3 would contain more hydrophobic compounds with longer retention times.

**Figure 3:**
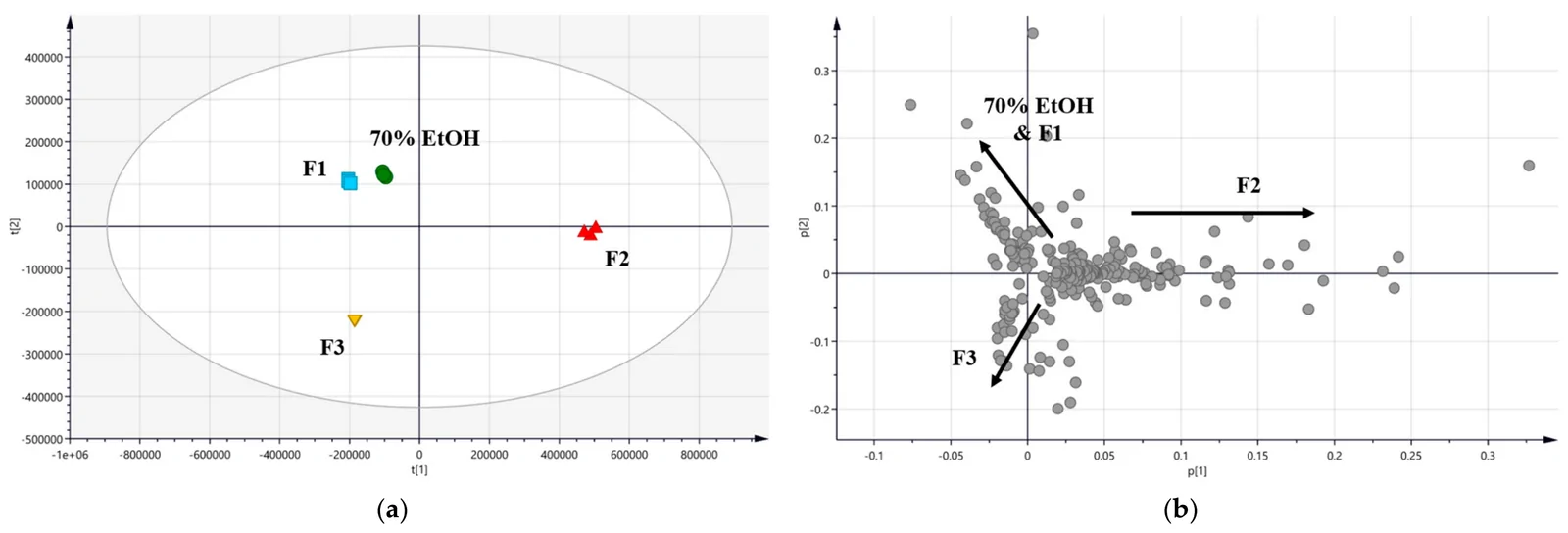
PCA score plot and loading scatter plot for *Glehnia littoralis* extract and its fractions in negative-ion mode. (**a**) PCA score plot. Fraction 2 was distinguished from Fraction 3, Fraction 1 and 70% ethanol extract by 76.6% variance for PC1. Fraction 3 was distinguished from Fraction 1 and 70% ethanol extract by 17.4% variance for PC2. (**b**) Loading scatter plot. Distinctive compounds that could have influence on PCA score plot between ethanolic extract and fractions were presented in loading scatter plot.

To identify metabolites that were differentially and characteristically present in F2—the fraction exhibiting the highest antioxidant, α-glucosidase, and DPP-IV inhibitory activities—multivariate statistical analysis was employed. An unsupervised Principal Component Analysis (PCA) was conducted on the negative-mode MS dataset to observe overall chemical clustering. In the negative-mode PCA score plot (Figure 3a), the raw extract and its fractions were clearly separated into distinct clusters. The two principal components explained 94.0% of the total variance, with PC1 accounting for 76.6% and PC2 explaining 17.4%. F2 was distinct from the extract and the other fractions based on PC1, while F3 was differentiated from the extract and F1 using PC2.

To identify the key variables driving this spatial segregation, a loading scatter plot was generated (Figure 3b). This plot revealed the presence of distinctive compounds in negative mode critical for segregating F2 from the other areas on the PCA score plots.

### 3.6. Biomarker Selection and Tentative Identification

To explore the discriminating metabolites concentrated in the active F2 fraction, an orthogonal partial least squares-discriminant analysis (OPLS-DA) model was subsequently established. To select key characteristic variables with high statistical confidence, the variables were filtered based on a Variable Importance in Projection (VIP) threshold of VIP ≥ 2.0 in negative ionization mode, together with *p*(corr), the correlation-scaled loading of the OPLS-DA model (S-plot), which ranges from −1 to 1. All selected features showed *p*(corr) values of 0.96–0.99, indicating a strong positive association with F2.

Among the screened metabolites satisfying these parameters, the major contributing compounds were selected and tentatively identified. Notably, two major flavonoid glycosides—rutin (*m*/*z* 609.1458) and quercetin 3-β-D-glucoside (Q3G, *m*/*z* 463.0879)—along with other significant components such as chlorogenic acid (*m*/*z* 353.0875) and kaempferol-3-*O*-rutinoside (*m*/*z* 593.1509), were selected as the key distinctive biomarkers elevated in the active F2 matrix. Several additional spectral features also satisfied the VIP ≥ 2.0 threshold (Table 2). Most of these, however, could not be structurally assigned with confidence based on available MS/MS fragmentation patterns and database matching, and were therefore labeled as unknown and excluded from further biological validation. A putative quinic acid derivative (*m*/*z* 191.0553) was tentatively identified but was not prioritized for downstream validation given its comparatively low VIP ranking relative to the four primary biomarkers.

The tentative identification parameters of these key biomarkers satisfying the VIP ≥ 2.0 threshold in negative ESI mode are summarized in Table 2. These exploratory findings suggest that the enzyme-inhibitory profile of the F2 fraction may be associated with these concentrated polyphenolic constituents.

### 3.7. Inhibitory Activities of the Marker Compounds

Rutin, Q3G, and chlorogenic acid (MSI Level 1) were evaluated as pure standards under the same assay conditions (Table 3) to verify whether the statistically selected markers themselves inhibit the enzymes and to obtain their IC_50_ values for the concentration-addition analysis. These values do not necessarily reflect their behavior within the extract matrix.

In the α-glucosidase assay, Q3G exhibited the highest inhibitory effect with an IC_50_ value of 170.68 ± 10.35 µg/mL (367.54 µM), which was significantly stronger than that of the conventional positive control, acarbose (IC_50_ = 422.16 ± 57.02 µg/mL; 653.91 µM). Rutin also demonstrated highly potent inhibition, with an IC_50_ value of 247.50 ± 16.79 µg/mL (405.39 µM), comparable to Q3G and likewise stronger than acarbose. In contrast, chlorogenic acid showed markedly weaker α-glucosidase inhibitory activity (IC_50_ > 1600 µg/mL).

In the DPP-IV inhibitory profiling, consistent with the α-glucosidase assay results, Q3G again demonstrated the most potent inhibitory effect among the tested metabolites, exhibiting an IC_50_ value of 65.61 ± 11.50 µg/mL (141.28 µM), closely followed by rutin (IC_50_ = 89.09 ± 11.50 µg/mL; 145.93 µM). Chlorogenic acid showed comparatively weaker, though still measurable, DPP-IV inhibitory activity (IC_50_ = 189.48 ± 11.51 µg/mL; 534.78 µM), approximately 2- to 3-fold higher than that of rutin and Q3G. All three compounds showed much higher IC_50_ values than the synthetic positive control sitagliptin (IC_50_ = 0.0062 ± 0.0006 µg/mL; 0.0153 µM).

### 3.8. Metabolites Quantification by PRM Mode

Quantitative analysis of the key validated active metabolites within the crude 70% EtOH extract and the enriched F2 fraction was performed using UHPLC-Orbitrap MS/MS in Parallel Reaction Monitoring (PRM) mode. In negative HESI mode, linear calibration curves were successfully generated using high-purity reference standards for chlorogenic acid (*m*/*z* 353.0878), rutin (*m*/*z* 609.1463), and quercetin 3-β-D-glucoside (*m*/*z* 463.0881).

Based on the PRM-based estimates, rutin was the most abundant of the quantified compounds in both the crude 70% EtOH extract (25.39 ± 0.87 mg/g dry weight) and the refined F2 fraction (188.97 ± 2.54 mg/g dry weight). Highly significant enrichments of all target biomarkers were achieved in F2 compared to the crude extract; notably, the concentration of the highly active quercetin 3-β-D-glucoside was enriched approximately 25-fold in F2 (43.44 ± 0.69 mg/g) relative to its initial level in the raw extract (1.75 ± 0.03 mg/g). Additionally, chlorogenic acid was also heavily concentrated in the F2 matrix (30.61 ± 0.04 mg/g) compared to the crude extract (5.79 ± 0.03 mg/g). This enrichment was consistent with the higher inhibitory activities of F2.

## 4. Discussion

The extraction and fractionation profiles of *Glehnia littoralis* leaves and stems revealed significant variations in yield and color characteristics depending on the polarity of the mobile phase, yielding a colorless hydrophilic fraction (F1), a slight yellow fraction (F2), and a green hydrophobic fraction (F3). The quantitative screening of bioactive compound classes demonstrated that the total phenolic content (TPC) and total flavonoid content (TFC) were highly concentrated in the F2 fraction, displaying 234.71 ± 0.31 mg GAE/g and 155.61 ± 6.99 mg QE/g, respectively, which were significantly higher than those of the crude 70% ethanol extract and other fractions. This dense concentration of polyphenolic structures in F2 correlates strongly with its superior antioxidant capability, as evidenced by the lowest observed IC_50_ value of 32.09 ± 2.52 µg/mL against DPPH free radicals, although the DPPH assay, as a chemical assay, does not demonstrate protection against oxidative stress in biological systems.

In the development of natural interventions for type 2 diabetes mellitus (T2DM), dual-targeting inhibition of key blood glucose-regulating enzymes remains a critical benchmark. In this study, F2 exhibited the most potent dual inhibitory efficacy, yielding an IC_50_ value of 314.41 ± 6.78 µg/mL against α-glucosidase and an IC_50_ value of 209.67 ± 3.74 µg/mL against DPP-IV. The α-glucosidase inhibition of F2 was comparable to that of acarbose (IC_50_ = 422.16 ± 57.02 µg/mL) under the present assay conditions. Yeast α-glucosidase is widely used for the in vitro screening of plant-derived inhibitors [20]; however, the inhibitory potency of acarbose is known to vary depending on the origin of the enzyme [28], and a wide range of IC_50_ values has been reported for acarbose against yeast α-glucosidase (e.g., 6.2 and 0.99 mg/mL) [29,30]. Therefore, the comparison with acarbose reflects relative potency within this screening model, and its relevance to mammalian intestinal α-glucosidase remains to be confirmed. Although its DPP-IV inhibition was lower than the synthetic drug sitagliptin, F2 inhibited both enzymes in vitro.

To explore the constituents potentially associated with this activity, exploratory non-targeted metabolomics coupled with multivariate statistical analysis (PCA and OPLS-DA) was conducted. The F2 matrix was clearly segregated on the PCA plot, explaining 94.0% of the total variance in negative mode (PC1: 76.6%, PC2: 17.4%). Using VIP ≥ 2.0 together with high positive *p*(corr) values (0.96–0.99), four compounds—rutin, chlorogenic acid, quercetin 3-β-D-glucoside (Q3G), and kaempferol-3-*O*-rutinoside—were selected as the primary distinctive biomarkers, with kaempferol-3-*O*-rutinoside in fact showing the second-highest VIP score among them. Despite this strong statistical contribution to class separation, rutin, Q3G, and chlorogenic acid were the only biomarkers identified at MSI Level 1 and consequently carried forward into pure-standard enzyme inhibition assays and PRM quantification. Kaempferol-3-*O*-rutinoside remained at the putative identification stage, based solely on accurate mass and database-matched MS/MS fragmentation, and its quantitative contribution to the bioactivity of F2 therefore remains to be confirmed in future studies.

Subsequent validation using pure standards confirmed that Q3G possesses the highest inhibitory potency among the individual compounds, with an IC_50_ of 170.68 ± 10.35 µg/mL for α-glucosidase (lower than acarbose on a molar basis) and 65.61 ± 11.50 µg/mL for DPP-IV. Rutin also displayed robust inhibitory activity, with IC_50_ values of 247.50 ± 16.79 µg/mL and 89.09 ± 11.50 µg/mL, respectively. PRM-based estimates indicated that rutin was the most abundant of the quantified compounds, at 188.97 ± 2.54 mg/g in F2, while Q3G was enriched approximately 25-fold in F2 (43.44 ± 0.69 mg/g) compared to the crude extract.

The relative contribution of rutin, Q3G, and chlorogenic acid to F2’s overall potency was estimated using a simplified concentration-addition (dose-additivity) model, similar in principle to combination-index approaches previously used to evaluate additive or synergistic interactions among phenolic compounds against α-glucosidase [31,32,33]. At F2’s IC_50_ for α-glucosidase (314.41 µg/mL), the estimated rutin, Q3G, and chlorogenic acid concentrations (59.41, 13.66, and 9.62 µg/mL, respectively) corresponded to dose fractions of 0.24, 0.08, and <0.01, respectively, with a sum of approximately 0.32. Similarly, at F2’s IC_50_ for DPP-IV (209.67 µg/mL), the dose fractions of rutin, Q3G, and chlorogenic acid were 0.44, 0.14, and 0.03, respectively, with a sum of approximately 0.62. As both summed fractions remained below 1, rutin, Q3G, and chlorogenic acid together could not fully account for the potency of F2. These dose fractions should be regarded as approximate, because the concentration-addition model assumes additive, non-interacting effects and the PRM-based contents are estimates that may be affected by matrix effects. This residual effect may be attributable to additional bioactive constituents present in the fraction that were not captured by the present quantitative validation—most notably kaempferol-3-*O*-rutinoside, which satisfied the same biomarker selection criteria (VIP ≥ 2.0, *p*(corr) = 0.99, with the second-highest VIP score among the four candidates) but remained putatively identified rather than confirmed against an authentic standard. Positive-mode features such as the furanocoumarin isopimpinellin (VIP 2.51) and matrix effects on the PRM-based estimates may also contribute. Isolating and validating this and other unconfirmed constituents represents an important direction for future work. Possible interactive effects among the biomarkers cannot be excluded either but confirming their precise nature would require dedicated combination-index or synergy assays using isolated standards, which were beyond the scope of the present study.

The superior α-glucosidase and DPP-IV inhibitory activities of rutin and Q3G identified in this study are consistent with previous reports on flavonoid glycosides. Rutin has been documented to exhibit potent α-glucosidase inhibitory activity with an IC_50_ value of 0.037 µM, which was stronger than quercetin, suggesting that glycosylation enhances the inhibitory capacity of flavonoids against carbohydrate-hydrolyzing enzymes [34]. Furthermore, rutin has demonstrated significant DPP-IV inhibitory activity with an IC_50_ value of 485 µM in citrus bioflavonoid studies, supporting its role as a multi-target antidiabetic agent [35]. Notably, Q3G itself has recently been identified as a key phenolic constituent governing α-glucosidase inhibition in apple-derived matrices, underscoring its relevance as a food-derived inhibitory agent independent of the aglycone quercetin [36].

Beyond enzyme inhibition, the antidiabetic potential of rutin has been extensively validated in animal models. Oral administration of rutin (100 mg/kg) for 45 days in streptozotocin-induced diabetic rats resulted in significant reductions in plasma glucose, increased insulin levels, and restoration of glycogen content and carbohydrate metabolic enzyme activities, accompanied by histopathological evidence of pancreatic islet protection [37,38]. These reports support further in vivo investigation of rutin-rich fractions such as F2.

When evaluated against Korea’s officially recognized blood glucose-regulating functional ingredients, such as guava leaves [39] and banaba leaves [40], the raw *Glehnia littoralis* extract itself shows insufficient phenolic content and crude activity. However, the prep-HPLC refined F2 fraction effectively bridges this performance gap, matching the phenolic capacity of guava leaf extract and offering a significantly higher enrichment of the high-potency biomarker Q3G. This indicates that the prep-HPLC refinement step provides a practical route to closing this performance gap, even though, as discussed above, rutin and Q3G together cannot fully account for F2’s potency on their own.

The enrichment strategy employed in this study also draws parallels with other halophyte-based antidiabetic research. For instance, *Salicornia herbacea*, a halophyte from the Chenopodiaceae family, has demonstrated anti-hyperglycemic effects in db/db mice and streptozotocin-induced diabetic rats, attributed to its flavonoid and phenolic acid constituents [41,42]. While *Glehnia littoralis* belongs to the Apiaceae family, the convergence of flavonoid glycoside-mediated mechanisms across diverse halophytic species underscores the therapeutic promise of salt-tolerant plants as sources of multi-target antidiabetic agents. More broadly, comparative surveys of Korean halophytes have highlighted flavonoid- and phenolic-rich species as consistent sources of antioxidant and antidiabetic activity across taxonomically diverse genera [43], reinforcing the premise that salt adaptation favors the accumulation of enzyme-inhibitory phenolic constituents in aerial plant tissues.

Collectively, these findings position the F2 fraction of *Glehnia littoralis* leaves and stems as a flavonoid-enriched fraction with in vitro α-glucosidase and DPP-IV inhibitory activities, to which rutin and Q3G appear to contribute in part, whereas chlorogenic acid appears to contribute only marginally.

This study has several limitations. Enzyme inhibition was evaluated only in vitro using yeast α-glucosidase; PRM quantification was performed without isotope-labeled internal standards or recovery experiments; and in the metabolomic analysis, the replicates were aliquots of the same extract, the injection order was not randomized, and pooled QC samples were not included. In addition, the activities of the pure standards may not reflect their behavior within the extract matrix, and the concentration-addition analysis assumes additive effects and relies on estimated PRM contents. Accordingly, the metabolomic and dose-fraction results should be regarded as exploratory. These points should be addressed in future studies, together with cell-based and in vivo evaluation.

## 5. Conclusions

In summary, this study systematically evaluated the in vitro α-glucosidase and DPP-IV inhibitory and antioxidant activities of the leaves and stems of *Glehnia littoralis*, which have long been consumed as a traditional food source (e.g., namul) yet remain comparatively understudied relative to its roots. Through targeted preparative high-performance liquid chromatography, the F2 fraction successfully concentrated the key polyphenolic and flavonoid components from the crude ethanolic extract, resulting in the most potent DPPH radical scavenging activity and dual-targeting enzyme inhibitory efficacy against both α-glucosidase and DPP-IV among the tested materials, with α-glucosidase inhibition comparable to that of acarbose in the yeast enzyme assay.

Exploratory non-targeted metabolomics coupled with PRM-based quantification indicated that rutin, quercetin 3-β-D-glucoside (Q3G), and chlorogenic acid were enriched in F2. A concentration-addition comparison between F2’s IC_50_ values and the individual potencies of these three compounds, however, showed that their summed dose fractions at F2’s IC_50_ were only approximately 0.32 and 0.62 for α-glucosidase and DPP-IV, respectively. This gap suggests that these three compounds explain only part of F2’s overall potency, possibly reflecting the presence of additional, as-yet unconfirmed constituents within the fraction. Possible interactive effects among the biomarkers cannot be excluded either, though confirming this would require dedicated combination-index or synergy assays beyond the scope of the present study.

While the crude extract requires further refinement to match existing commercial materials, the refined F2 fraction warrants further investigation as a potential functional food resource for blood glucose regulation. Given that rutin and Q3G, as glycosylated flavonoids, require intestinal deglycosylation to their aglycone (quercetin) prior to absorption, whereas quercetin, the aglycone form, can be absorbed more directly via passive diffusion in the stomach and small intestine [44,45], future in vivo studies should also address the pharmacokinetic fate of these biomarkers to clarify their physiological relevance beyond the in vitro enzymatic assays employed here. Future work should focus on isolating and validating additional contributing constituents, directly testing combination effects among the key biomarkers, and exploring deeper mechanisms—including cellular glucose uptake, restoration of insulin sensitivity, and protection of pancreatic β-cell function—as well as subsequent animal and human application trials.

## Figures and Tables

**Table 1:** Total phenolic content (TPC), total flavonoid content (TFC), and DPPH radical scavenging activity (IC_50_) of the *Glehnia littoralis* 70% ethanol extract and its prep-HPLC fractions (F1, F2, and F3).

Sample	Total Phenolic Content (mg GAE/g of Dry Weight of Sample)	Total Flavonoid Content (mg QE/g of Dry Weight of Sample)	IC_50_ (µg/mL) of DPPH Free Radical Scavenging Activity
**70% EtOH**	38.43 ± 0.66 ^c^	21.47 ± 1.09 ^b′^	203.99 ± 4.25 ^c″^
**F1**	22.58 ± 0.45 ^d^	6.67 ± 0.15 ^c′^	425.24 ± 17.72 ^d″^
**F2**	234.71 ± 0.31 ^a^	155.61 ± 6.99 ^a′^	32.09 ± 2.52 ^b″^
**F3**	48.56 ± 1.04 ^b^	20.44 ± 1.11 ^b′^	463.91 ± 4.68 ^e″^
**Ascorbic acid**	-	-	4.01 ± 0.06 ^a″^

The results are based on three independent replicates and expressed as the mean ± standard deviation (SD); Different letters denote significant differences (*p* < 0.05).

**Table 2:** Tentatively identified active biomarkers in F2 fraction of *Glehnia littoralis* with VIP ≥ 2.0 in negative-ion (NEG) mode.

*m*/*z*	RT (min)	Tentative Identification	MS/MS Fragment	VIP	*p* (corr)	Sub Class	Error (ppm)
609.1458	9.33	Rutin	300.0274, 301.0345, 271.0247	5.19	0.96	Flavonoid glycosides	−0.49
593.1509	9.59	kaempferol-3-*O*-rutinoside	285.0400, 284.0325, 255.0297	3.85	0.99	Flavonoid glycosides	−0.53
609.1458	9.73	Rutin isomer	300.0276, 301.0349, 271.0248	3.81	0.99	Flavonoid glycosides	−0.49
353.0875	8.12	Chlorogenic acid	191.0555, 173.0448, 135.0442	3.69	0.97	Quinic acid derivatives	−0.81
481.1383	10.41	Unknown	96.9589, 437.1484, 395.1379	3.07	0.99	-	-
463.0879	9.50	Quercetin 3-β-D-glucoside	300.0274, 271.0247, 301.0344	2.93	0.98	Flavonoid glycosides	−0.48
659.2719	10.47	Unknown	295.1087, 253.0618, 299.1037	2.87	0.98	-	-
353.0876	8.55	Chlorogenic acid isomer	191.0554, 173.0448, 135.0442	2.69	0.99	Quinic acid derivatives	−0.44
659.2721	10.76	Unknown	295.1088, 253.0617, 299.1036	2.51	0.96	-	-
610.1489	9.74	Rutin derivatives	300.0275, 301.0339, 271.0248	2.09	0.99	Flavonoid glycosides	-
481.1383	10.63	Unknown	96.9589, 437.1485, 395.1380	2.09	0.99	-	-
191.0553	8.11	D-(-)-Quinic acid	191.0554, 85.0283, 127.0391	2.08	0.99	Quinic acid derivatives	−4.04
517.1019	10.09	Unknown	96.9589, 241.0020, 517.1021	2.06	0.99	-	-
721.3651	13.23	Unknown	277.2173, 59.0127, 89.0233	2.06	0.98	-	-

*m*/*z* values were obtained from non-targeted full-scan (MS^1^) feature detection (Compound Discoverer 3.1) and may differ by <1 ppm from the PRM-based *m/z* values reported in Section 2.9 for the same compounds, reflecting differences in acquisition mode rather than compound misidentification. MSI level: rutin, Q3G, and chlorogenic acid, Level 1; other annotated compounds, Level 2; unknown, Level 4. *p* (corr), correlation-scaled loading of the OPLS-DA model (S-plot); it is not a statistical significance level.

**Table 3:** IC_50_ values of pure standards of the marker compounds identified in F2 and the positive controls.

Compounds	IC_50_ (µM) of α-Glucosidase Inhibition	IC_50_ (µM) of DPP-IV Inhibition
Chlorogenic acid	>4515.82	534.78 ± 32.47 ^c′^
Rutin	405.39 ± 27.50 ^a^	145.93 ± 18.84 ^b′^
Quercetin 3-β-D-glucoside	367.54 ± 22.29 ^a^	141.28 ± 24.77 ^b′^
Acarbose	653.91 ± 88.32 ^b^	-
Sitagliptin	-	0.0153 ± 0.0014 ^a′^

The results are based on three independent replicates and expressed as the mean ± standard deviation (SD); IC_50_ values were determined by probit analysis; Different letters denote significant differences (*p* < 0.05).

## Data Availability

The data used to support the findings of this study can be made available by the corresponding author upon request.

## Supplementary Materials

The following supporting information can be downloaded at: https://www.mdpi.com/article/10.3390/foods15213980/s1. Figure S1: PCA score plot and loading scatter plot for *Glehnia littoralis* extract and its fractions in positive-ion mode. (a) PCA score plot. Fraction 2 was distinguished from Fraction 3, Fraction 1 and 70% ethanol extract by 64.6% variance for PC1. Fraction 3 was distinguished from Fraction 1 and 70% ethanol extract by 20.2% variance for PC2. (b) Loading scatter plot. Distinctive compounds that could have influence on PCA score plot between ethanolic extract and fractions were presented in loading scatter plot.

## Abbreviations

The following abbreviations are used in this manuscript:

ANOVA	Analysis of Variance
DAD	Diode Array Detector
DMSO	Dimethyl Sulfoxide
DPP-IV	Dipeptidyl Peptidase-IV
DPPH	2,2-Diphenyl-1-picrylhydrazyl
EP	Extra Pure
ESI	Electrospray Ionization
EtOH	Ethanol
GAE	Gallic Acid Equivalent
HESI	Heated Electrospray Ionization
IC_50_	Half-Maximal Inhibitory Concentration
LC	Liquid Chromatography
MS	Mass Spectrometry
MSI	Metabolomics Standards Initiative
NEG	Negative Ionization
OPLS-DA	Orthogonal Partial Least Squares-Discriminant Analysis
PC1	Principal Component 1
PC2	Principal Component 2
PCA	Principal Component Analysis
pNPG	4-Nitrophenyl α-D-glucopyranoside
POS	Positive Ionization
prep-HPLC	Preparative High-Performance Liquid Chromatography
PRM	Parallel Reaction Monitoring
PVDF	Polyvinylidene Fluoride
Q3G	Quercetin 3-β-D-glucoside
QC	Quality Control
QE	Quercetin Equivalent
RT	Retention Time
SD	Standard Deviation
S/N	Signal-to-Noise Ratio
T2DM	Type 2 Diabetes Mellitus
TFC	Total Flavonoid Content
TIC	Total Ion Chromatogram
TPC	Total Phenolic Content
UHPLC	Ultra High-Performance Liquid Chromatography
VIP	Variable Importance in Projection
