# Supplementary material for: In Vitro α-Glucosidase and Dipeptidyl Peptidase-IV Inhibitory Activities of Ethanol Extract and Its Fractions from the Leaves and Stems of Glehnia littoralis
Source: Foods. 2026 Nov 6;15(21):3980. doi: 10.3390/foods15213980 (PMC13762584; doi:10.3390/foods15213980)
Supplement: Supplementary file 1 [file foods-15-03980-s001.zip › foods-4609571-supplementary.pdf]

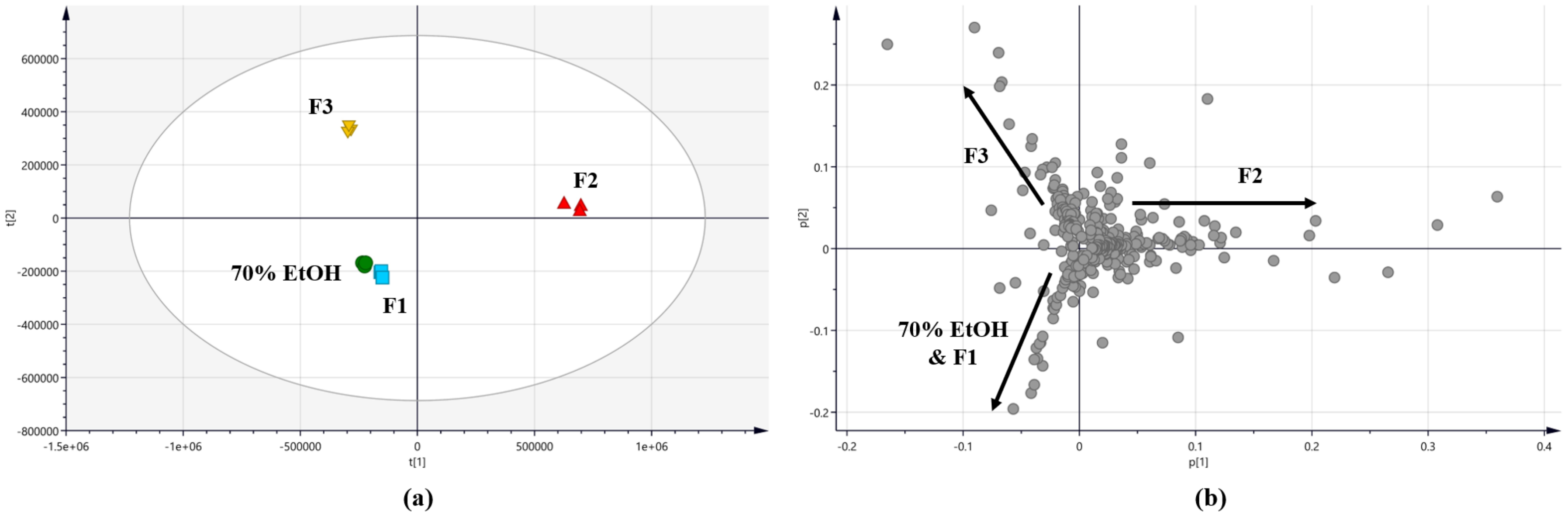

Figure S1. PCA score plot and loading scatter plot for *Glehnia littoralis* extract and its fractions in positive-ion mode. (a) PCA score plot. Fraction 2 was distinguished from Fraction 3, Fraction 1 and 70% ethanol extract by 64.6% variance for PC1. Fraction 3 was distinguished from Fraction 1 and 70% ethanol extract by 20.2% variance for PC2. **(b) Loading scatter plot.** Distinctive compounds that could have influence on PCA score plot between ethanolic extract and fractions were presented in loading scatter plot.
